# Health-related quality of life in children and adolescents with congenital diaphragmatic hernia: a cross-sectional study

**DOI:** 10.1186/s12955-018-0869-z

**Published:** 2018-03-14

**Authors:** Katarina Bojanić, Ruža Grizelj, Jurica Vuković, Lana Omerza, Marina Grubić, Tomislav Ćaleta, Toby N. Weingarten, Darrell R. Schroeder, Juraj Sprung

**Affiliations:** 1 Division of Neonatology, Department of Obstetrics and Gynecology, University Hospital Merkur, Zagreb, Croatia; 2 Department of Pediatrics, School of Medicine, University of Zagreb, University Hospital Centre, Zagreb, Croatia; 3 Division of Multispecialty Anesthesia, Mayo Clinic, 200 First Street SW, Rochester, MN 55905 USA; 4 Division of Biomedical Statistics and Informatics, Mayo Clinic, Rochester, MN USA

**Keywords:** Congenital diaphragmatic hernia, Survivors, Health-related quality of life, Cross-sectional studies

## Abstract

**Background:**

Patients with congenital diaphragmatic hernia (CDH) have a high residual morbidity rate. We compared self-reported health-related quality of life (HRQoL) between patients with CDH and healthy children.

**Methods:**

Forty-five patients with CDH who were born from January 1, 1990, through February 15, 2015, were matched to healthy, age-matched control participants at a 1:2 ratio. The health records of the study participants were reviewed to determine comorbid conditions, and HRQoL was assessed by both the participants and their parents with the Pediatric Quality of Life Inventory (PedsQL). The HRQoL scores of the patients with CDH and the control participants were compared by using analysis of variance to adjust for age group and sex. Among patients with CDH, analysis of variance was used to compare HRQoL scores across groups defined according to their characteristics at initial hospitalization, postdischarge events, and comorbid conditions.

**Results:**

Compared with control participants, patients with CDH had lower mean PedsQL scores, as reported by the parent and child, for the physical and psychosocial domains (*P* < 0.001). Risk factors associated with lower parent-reported HRQoL included bronchopulmonary dysplasia, longer initial hospitalization, severe cognitive impairment, and orthopedic symptoms; among patients with CDH, low HRQoL was associated with chronic respiratory issues.

**Conclusion:**

Patients with CDH had lower HRQoL compared with healthy participants. Parent-reported HRQoL tended to be higher than child-reported HRQoL. Results were also inconsistent for the risk factors associated with HRQoL obtained by using child- and parent-reported scores. Therefore, when interpreting HRQoL in CDH survivors, a proxy report should not be considered a substitute for a child’s self-report.

## Background

Assessment of chronically ill children and adolescents with quality of life (QoL) instruments provides subjective information about well-being in various domains of daily life. Better understanding of health-related QoL (HRQoL) of these patients may enable health practitioners to better understand disease-specific symptoms, their association with psychosocial functioning, and the development in the daily life of children [1, 2]; this may ultimately help clinical decision-making and parent counseling [3–5]. Bochdalek congenital diaphragmatic hernia (CDH) is a rare developmental defect of the diaphragm that causes the abdominal viscera to herniate into the chest. Patients with CDH have high morbidity and mortality rates [6, 7]. CDH manifests when the diaphragm fails to close during development, and the abdominal contents migrate into the chest through this defect; pulmonary hypoplasia results because there is limited room for the lungs to grow. These patients often have pulmonary hypertension, asthma, gastrointestinal reflux disease, feeding disorders, developmental delays, and other comorbid conditions that may persist into adulthood [8–15]. This poor health status can substantially affect QoL. Few studies have described HRQoL in patients with CDH [14, 16–19]. One study reported that 6- to 16-year-old patients with CDH have lower HRQoL than similarly aged healthy children [14]. Michel et al. [18] reported considerably lower HRQoL scores for patients with CDH who were 2.6 to 13.8 years old than for age- and sex-matched control participants. In a cross-sectional study that included patients born from 1969 through 1996, Poley et al. [19] found that these differences in HRQoL between patients with CDH and healthy control patients decreased with age. However, given the cross-sectional nature of that study, the enrolled patients were born when the primary treatment of CDH was considerably different than current treatment; therefore, differences among age groups could be due to survival bias or changes in treatment modalities. This rare patient population is most commonly examined using cross-sectional studies, and contemporary cohorts consisting of patients with CDH born after the introduction of new therapeutic modalities (eg, protective ventilation, extracorporeal membrane oxygenation [20–22]) are needed.

Our institution is a tertiary referral center in Croatia for patients with CDH. Our institutional registry of patients with CDH provides the opportunity to study patients with CDH, and describe temporal effects of CDH on physical and mental function [6–9, 23, 24]. The primary objective of the current cross-sectional study was to compare HRQoL between patients with CDH and a control group of similarly aged, healthy children. The Pediatric Quality of Life Inventory (PedsQL) [25], a generic HRQoL questionnaire, is one of the most commonly used instruments for the assessment of physical, mental, and social domains in healthy persons and persons with functional limitations [26, 27]. We hypothesized that PedsQL scores would be significantly lower for CDH survivors compared to healthy controls. The secondary objective was to examine potential associations among CDH characteristics at initial presentation (eg, severity of presentation at birth), postdischarge events, and HRQoL scores of patients with CDH. QoL is not only a measure of health but also reflects health perceptions and expectations, which may be influenced by physical, psychological, and socioeconomic characteristics. Because PedsQL allows assessment of the child self-report and the parent-proxy report, and because of the inherent subjectivity of assessing QoL, our tertiary objective was to evaluate cross-informant variance in HRQoL ratings between parents and their children with CDH. We aimed to determine whether the proxy report could be used as a substitute for the child’s self-report for assessing QoL in patients with CDH.

## Methods

This study was approved by the Institutional Ethics Committee of University Hospital Centre in Zagreb, Croatia. All procedures were conducted in accordance with the ethical standards of the institutional or national research committee, the 1964 Helsinki Declaration and its later amendments, or comparable ethical standards. Written informed consent was obtained from all participants or their legal guardians.

### Study design and participants

#### Patients with CDH

This cross-sectional, single-center study of patients with Bochdalek CDH was conducted at University Hospital Centre, which is the largest Croatian medical center for neonatal care. All patients with CDH born from January 1, 1990, through February 1, 2015, were invited to participate, and their parents received a telephone call or letter in which the study objectives were described. HRQoL questionnaires were completed by the patients and their parents from March 1, 2015, through September 30, 2015.

#### Control participants

Patients with CDH were matched with healthy control participants at a 1:2 ratio. Control participants were recruited during well-child visits or visits for acute and minor illnesses. Inclusion criteria were similar age to the patients with CDH and no known chronic medical illness. This approach to recruit control participants by excluding those with chronic conditions has been utilized before [19]. Our control participants and their parents completed the HRQoL questionnaires during the same time frame as the patients with CDH. Except for sex and age, no demographic information was collected about the control participants.

### Data collection and interviews

In accordance with the original PedsQL administration guidelines, questionnaires were completed by the participants and their parents before the completion of other health data forms or the child’s physical examination. Parents or participants independently completed the questionnaires without consultation.

### HRQoL evaluation

We obtained permission to use the PedsQL questionnaire (version 4.0) [25]. This instrument has been validated to evaluate healthy children and adolescents, as well as patients with acute and chronic health conditions [26, 27]. The PedsQL questionnaire provides a child self-report (for children ≥5 years) and a parent-proxy report (for children > 2 years). Therefore, for children younger than 5 years, only their parents completed the age-appropriate HRQoL forms. For infants 1 to 12 and 13 to 24 months old, we obtained parent-proxy reports by using the PedsQL infant scales. Infant Scales, include 36 items for 1 to 12-month-old infants and 45 items for 13 to 24-month-old children that assess 5 dimensions (physical, emotional, social, and cognitive functioning and physical symptoms). For 2 to 4-year-old children, we used the PedsQL Parent Report for Toddlers, which includes 21 items that assess 4 dimensions (physical, emotional, social, and preschool functioning). For the children ages 5 to 7, 8 to 12, and 13 to 18 years old, and young adults older than 18 up to 25 years old (PedsQL Young Adult Version) the self-report scales include 23 items, which differ slightly according to age group, that assess 4 dimensions (physical, emotional, social, and school functioning). Parents and older children (≥5 years) self-administered PedsQL after receiving instructions. However, for 5 to 7-year-old children, PedsQL was administered by reading the instructions to them (each item was read word-for-word) and providing a separate page that showed 3 facial expressions (ie, pictorial representations) to help the child understand how to answer. Children aged 8 years and older scored questions using a 5-point Likert scale (0 [never] to 4 [almost always]), and 5- to 7-year-old children used a 3-point Likert scale anchored to the pictorial representations.

Although the PedsQL questionnaire introduces various items (ie, questions) for different ages, the forms, regardless of age group, are essentially identical and only differ in the developmentally appropriate language that is used. Each response receives a value of 0%, 25%, 50%, or 100% (representing a range from “never” to “always”) [25]. The sum of the items divided by the number of answered items generates the mean score. All responses were tabulated to generate an overall health score and physical health summary score. A psychosocial health summary score was calculated using the sum of the answered items in the emotional, social, school, and cognitive (or school) function domains; separate scores were also calculated for each of these subdomains in accordance with published recommendations [25].

### Perinatal health information and childhood medical history

After completion of PedsQL, all patients (or their parents) were interviewed to obtain their medical and surgical histories. This interview was conducted by a neonatologist (K.B.). In addition, participant health records were reviewed to determine their demographic, neonatal, and postnatal characteristics and medical histories. The collected perinatal variables were sex, gestational age, birth weight, postnatal duration of mechanical ventilation, duration of hospitalization, history of bronchopulmonary dysplasia (BPD) (as defined by Jobe and Bancalari) [28], type of CDH in regard to the liver position (“liver up” vs “liver down,” describes whether liver was herniated [“up”] or not [“down”] into the thoracic cavity), and congenital anomalies. By using birth weight and Apgar score at 5 min, we calculated probability of survival scores as proposed by the Congenital Diaphragmatic Hernia Study Group (Apgar score assesses 5 vital signs—appearance, pulse, grimace, activity, respiration—at 1 and 5 min after birth, with the final score ranging from 0–10) [29]. This score assigns the probability of survival after birth, and neonates are categorized as having low (0%–33%), intermediate (34%–66%), or high (67%–100%) survival probability. Of note, this score is the accepted tool for grading CDH severity after birth. A recurrent respiratory infection (RRI) was defined as at least 3 respiratory episodes per year (or per fall-winter season) for at least 2 years and included treatment for wheezing and breathlessness [30]. Gastroesophageal reflux disease was recorded when indicated in the health records or when the patient received treatment. Furthermore, patients with CDH and their parents were interviewed to obtain information about current and chronic clinical issues using an inventory that was partly adopted from Michel et al. [18]. We considered 4 major health categories: digestive, respiratory, neuropsychological, and orthopedic. Digestive issues included oral aversion and chronic problems (eg, vomiting, reflux, constipation, diarrhea, abdominal pain, history of gastrointestinal surgery). Chronic respiratory issues included dyspnea at rest, exertional dyspnea, nocturnal dyspnea, chest pain, RRI, use of respiratory medications, and hospitalization for respiratory illness. Neuropsychological issues included cerebral palsy and major cognitive impairment (all patients were formally tested for neurocognitive function, and the results were previously reported [9]). Orthopedic issues included skeletal deformities or any orthopedic treatment. Finally, patients and their parents were asked about all hospital readmissions (following the initial hospitalization for CDH treatment) and surgical history.

### Data management

Parent- and child-reported HRQoL scores were compared between patients with CDH and control participants. Analysis of variance was used to adjust for sex and PedsQL questionnaire age group (infants: 1–12 months; infants: 13–24 months; toddlers: 2–4 years; young children: 5–7 years; children: 8–12 years; teens: 13–18 years; and young adults: 18–25 years). For these analyses, the age- and sex-adjusted differences between groups (patients with CDH vs control participants) are calculated using a multivariate regression mode with differences between groups (CDH vs control) presented for all scores using model estimates along with the corresponding 95% CI. Overall HRQoL scores were compared between parents and children using the paired *t* test when both assessments were available. To assess whether differences between parent-proxy report and child self-report were dependent on the age of the child, a repeated measure analysis of variance was performed with respondent (parent vs child), age group, and the respondent-by-age group interaction term included as the explanatory variables. In addition, in analyses restricted to CDH survivors and their parents, the association of HRQoL scores with CDH characteristics at initial hospitalization, postdischarge events, and comorbid conditions, was assessed using analysis of variance. Separate analyses were performed for parent proxy reports, and child self-reports. In order to create groups of sufficient size for meaningful comparisons when analyzing age at testing, the two infant groups (< 12 months, 13–24 months) were combined, as were children 5 to 7 and 8 to 12 years of age, and those 13 years of age and older. The analysis of child self-reports include only those ≥5 years of age at the time of testing since only parent reports were available for infants and children < 5 years old. Multiple comparison adjustments were not performed, and in all cases unadjusted 2-tailed *P* values are reported.

## Results

### Study population

**Fig. 1:**
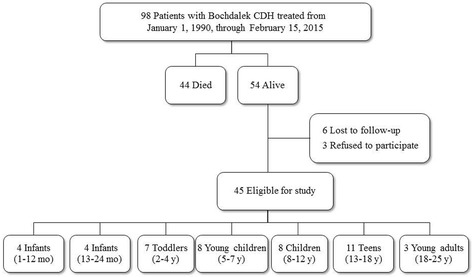
Exclusion and Enrollment of Patients With Bochdalek Congenital Diaphragmatic Hernia Treated at University Hospital Centre in Zagreb, Croatia. Abbreviations: CDH, congenital diaphragmatic hernia; mo, months; y, years

A total of 98 neonates with CDH were treated at University Hospital Centre from 1990 through 2015, and 44 neonates were known to have died at study recruitment (Fig. 1). Of the remaining 54 patients, 45 agreed to participate, 3 declined, and 6 were lost to follow-up. Therefore, in the current study, we examined self-reported HRQoL in 45 patients with CDH and 90 control participants. Patients with CDH had a mean (SD) age of 8.2 (5.7) years, and 29 patients (64%) were male. The number of CDH patients included in each of the PedsQL version– specific age groups is presented in the Fig. 1. The mean (SD) age of the control participants was 8.4 (6.1) years, and 40 participants (44%) were male.

### Comorbid conditions in patients with CDH

Among patients with CDH, 39 had left-sided CDH and 6 had right-sided CDH; 12 patients were prenatally diagnosed. Additional demographic, disease, and immediate postnatal surgical characteristics of these patients are shown in Table 1. The burden of these residual comorbid conditions was substantial (see the patient summary in the Appendix). Thirty-three patients with CDH (73%) were readmitted to the hospital at least 1 time (115 total readmissions) following their initial hospital discharge. The most common reason for subsequent hospitalization was related to respiratory problems (62 readmissions, of which the majority were for RRI), followed by digestive problems (26 readmissions). The remaining 27 readmissions were for a variety of reasons.

**Table 1:** Demographic, Disease, and Surgical Characteristics of Patients With CDH at Birth (*n* = 45)

Characteristic	Value
Prenatal diagnosis, n (%)	12 (26.7)
Male sex, n (%)	29 (64.4)
Gestational age, mean (SD), weeks	38.7 (2)
Birth weight, mean (SD), kg	3.11 (0.53)
Apgar score at 5 min, mean (SD)	7.8 (2.2)
Probability of survival, n (%)	
High (67%–100%)	31 (68.9)
Intermediate (34%–66%)	12 (26.7)
Low (0%–33%)	2 (4.4)
CDH side, n (%)^a^	
Left	39 (86.7)
Right	6 (13.3)
Duration of hospitalization, median (IQR), days	28 (18–57)
Congenital anomalies, n (%)	25 (55.6)
Cardiovascular^b^	7 (15.6)
Renal^c^	4 (8.9)
Gastrointestinal^d^	4 (8.9)
Skeletal^e^	7 (15.6)
Other^f^	3 (6.7)

*CDH* Congenital diaphragmatic hernia, *IQR* Interquartile range, *SD* Standard deviation

^a^ Seven patients with left-sided CDH were “liver up”; all 6 patients with right-sided CDH were “liver up”

^b^ Includes patent foramen ovale (*n* = 1), atrial septal defect II (*n* = 3), and patent ductus arteriosus (*n* = 3)

^c^ Includes hydronephrosis (*n* = 2), multicystic kidney (*n* = 1), and hypoplastic kidney (*n* = 1)

^d^ Includes accessory spleen (*n* = 2) and Meckel diverticulum (*n* = 2)

^e^ Includes foot deformities (*n* = 3) and abnormal number of ribs (*n* = 4)

^f^ Includes macroglossia (*n* = 1), neck fistula (*n* = 1), and preauricular tag (*n* = 1)

At the time of HRQoL testing, 29 patients with CDH (64%) reported recent respiratory issues, of which RRI was the most common (25 patients [56%]). Of these 25 patients with RRI, 9 (36%) had a history of BPD (6 patients had severe to moderate BPD, and 3 patients had mild BPD). Interestingly, 6 patients with BPD did not report a recent RRI (all patients had mild BPD and were > 10 years).

Twenty-three patients (51%) had digestive issues. Constipation was the most commonly reported problem (23 patients [51%]), followed by recurrent diarrhea and gastroesophageal reflux disease (each reported by 5 patients [11%]). Orthopedic issues affected 6 patients (13%), including 1 patient with pectus excavatum and restrictive pulmonary disease and 1 patient with kyphoscoliosis that required bracing therapy. Neurologic issues were identified in 7 patients with CDH (16%); all of these patients had severe cognitive impairment (including 1 patient with cerebral palsy).

### PedsQL scores of patients with CDH and control participants

Table 2 shows the parent- and child-reported HRQoL scores of the patients with CDH and control participants. Patients with CDH had significantly lower HRQoL (as reported by the parent-proxy report and patient self-report) compared with control participants, as shown by the overall scores, physical health summary scores, and psychosocial health summary scores (all *P* < 0.001) and the scores on the emotional, social, and school subdomains (all *P* ≤ 0.02). The only nonsignificant finding between control participants and patients with CDH was the parent-reported cognitive scores for 1- to 24-month-old children (*P* = 0.69). In the analysis restricted to complete child and parent pairs (ie, both completed HRQoL assessments [28 patients with CDH and 60 control participants]), the child’s self-reported scores were significantly lower than the parent-reported scores. The mean (SD) overall scores were 75.0 (13.9) for patients with CDH and 80.6 (12.0) for their parents (*P* = 0.046). The difference between parent and child reports was not found to be dependent on the child’s at testing (respondent-by-age interaction *P* = 0.769). The mean (SD) overall scores were 85.4 (8.8) for control participants and 88.8 (6.8) for their parents (*P* < 0.001), with no evidence of an interaction between respondent and age at testing (respondent-by-age interaction *P* = 0.533).

**Table 2:** Parent- and Child-Reported PedsQL Scores of Patients With CDH and Control Participants^a^

	Participants With CDH		Healthy Control Participants		Estimated Difference (CDH–Control)^b^		
Assessment	n	Mean Score (SD)	n	Mean Score (SD)	Estimate	95% CI	*P* Value
Parent report							
Overall score	45	81.2 (14.8)	90	89.2 (6.4)	−8.2	−11.7 to − 4.7	< 0.001
Physical health summary	45	82.2 (17.0)	90	90.5 (8.4)	−8.3	−12.5 to −4.1	< 0.001
Psychosocial health summary	45	80.1 (15.4)	90	88.5 (6.9)	−8.5	−12.2 to −4.7	< 0.001
Emotional	45	75.8 (15.2)	90	83.1 (11.0)	−8.0	−12.5 to −3.5	< 0.001
Social	45	85.6 (19.5)	90	93.2 (8.8)	−7.3	−11.9 to −2.8	0.002
School	37	79.3 (21.2)	74	91.0 (9.3)	−11.5	−17.2 to −5.8	< 0.001
Cognitive^c^	8	90.6 (11.1)	16	92.8 (9.9)	−1.8	−10.8 to 7.2	0.69
Child report							
Overall score	28	75.0 (13.9)	60	85.4 (8.8)	−10.7	−15.6 to − 5.9	< 0.001
Physical health summary	28	74.7 (16.5)	60	84.8 (11.6)	−10.7	− 16.7 to −4.7	< 0.001
Psychosocial health summary	28	75.4 (14.6)	60	85.6 (8.9)	−10.3	− 15.4 to −5.3	< 0.001
Emotional	28	69.6 (16.6)	60	79.8 (13.5)	−10.8	−17.4 to −4.2	0.002
Social	28	82.7 (18.3)	60	89.9 (9.9)	−6.9	−12.9 to − 1.0	0.02
School	28	73.2 (19.6)	60	86.8 (10.9)	−13.9	−20.4 to −7.4	< 0.001

*CDH* Congenital diaphragmatic hernia, *PedsQL* Pediatric Quality of Life Inventory, *SD* Standard deviation

^a^ Patients with CDH had a mean (SD) age of 8.2 (5.7) years and consisted of 29 males (64%) and 16 females (36%). Control participants had a mean (SD) age of 8.4 (6.1) years and consisted of 40 males (44%) and 50 females (56%)

^b^ The difference between patients with CDH and control was estimated using multiple regression with group (CDH vs. control) as the explanatory variable of interest and age group and sex included as adjustor variables

^c^Only reported by the parents of 1- to 24-month-old infants. For this age group, the psychosocial health summary score is calculated as the sum of the items divided by the number of items answered in the emotional, social, and cognitive functioning scales

Tables 3 and 4 present the HRQoL scores of the patients with CDH according to their characteristics at birth (including CDH severity) and after their initial hospital discharge. Among patients with CDH, these exploratory analyses showed that parent-reported HRQoL was lower when their children also had BPD, longer initial hospitalization, severe neurologic issues (most commonly severe cognitive impairment), or orthopedic symptoms; in addition, parent-reported HRQoL differed by the child’s age, and 5- to 12-year-old patients had the lowest HRQoL (Table 3). Among patients with CDH, a recent history of pulmonary symptoms was the only characteristic that was negatively associated with HRQoL (Table 4).

**Table 3:** Parent-Reported Quality of Life Scores of Patients With CDH Stratified by Characteristics at Birth, CDH Severity, and Major Lifetime Comorbid Conditions

Characteristic	Patients (n)	Mean Overall Score (SD)	*P* Value*	Mean Physical Score (SD)	*P* Value*	Mean Psychosocial Score (SD)	*P* Value*
Characteristics at birth							
Sex			0.38		0.72		0.35
Male	29	82.7 (15.4)		82.9 (18.3)		81.7 (16.0)	
Female	16	78.5 (13.5)		81.0 (14.9)		77.1 (14.5)	
Probability of survival score			0.08		0.06		0.08
Low or intermediate	14	75.5 (18.1)		75.0 (20.1)		74.0 (18.4)	
High	31	83.7 (12.5)		85.4 (14.7)		82.8 (13.3)	
CDH type			0.81		0.94		0.62
Liver down	32	80.8 (13.4)		82.1 (15.7)		79.3 (14.3)	
Liver up	13	82.1 (18.3)		82.5 (20.7)		81.9 (18.5)	
BPD			0.003		0.01		0.007
Yes	15	72.2 (19.2)		73.3 (20.3)		71.5 (20.0)	
No	30	85.7 (9.5)		86.7 (13.4)		84.4 (10.5)	
Initial hospitalization, days			0.01		0.15		0.008
≤ 30	24	86.9 (8.3)		86.1 (14.1)		86.4 (8.6)	
31–60	11	77.4 (10.3)		81.4 (16.3)		75.0 (10.1)	
≥ 61	10	71.3 (23.7)		73.7 (22.3)		70.4 (24.9)	
Mechanical ventilation, days			0.08		0.07		0.12
≤ 7	22	86.6 (8.8)		88.5 (12.3)		85.4 (9.4)	
8–21	13	78.4 (13.0)		78.7 (15.6)		76.8 (14.1)	
≥ 22	9	76.6 (20.0)		75.2 (23.3)		77.1 (19.6)	
Postdischarge events							
Hospitalizations, n			0.08		0.03		0.13
0	12	86.8 (10.0)		88.3 (9.5)		85.8 (12.1)	
1–2	17	83.1 (10.2)		86.3 (15.1)		81.5 (10.0)	
≥ 3	16	74.9 (19.6)		73.3 (20.2)		74.3 (20.5)	
Characteristics at testing							
Age			0.03		0.045		0.03
< 24 months	8	83.8 (9.2)		83.9 (7.8)		83.7 (11.3)	
2–4 years	7	93.2 (5.8)		97.3 (4.9)		90.7 (7.3)	
5–12 years	18	74.5 (17.2)		76.4 (19.0)		72.5 (18.2)	
≥ 13 years	12	82.4 (13.1)		81.0 (18.5)		82.8 (12.2)	
Digestive issues			0.06		0.046		0.07
Yes	23	77.2 (17.2)		77.3 (19.3)		76.0 (17.9)	
No	22	85.4 (10.5)		87.3 (12.7)		84.3 (11.3)	
Respiratory issues			0.40		0.13		0.62
Yes	29	79.8 (15.7)		79.3 (17.8)		79.2 (16.3)	
No	16	83.7 (13.0)		87.4 (14.7)		81.6 (14.1)	
Orthopedic issues			0.01		0.007		0.04
Yes	6	67.0 (21.9)		65.1 (23.4)		68.1 (22.1)	
No	39	83.4 (12.3)		84.8 (14.5)		81.9 (13.6)	
Neurologic issues			< 0.001		0.05		< 0.001
Yes^a^	7	64.7 (22.6)		70.8 (24.3)		60.4 (21.1)	
No	38	84.2 (10.7)		84.3 (14.8)		83.7 (11.1)	

*BPD* Bronchopulmonary dysplasia, *CDH* Congenital diaphragmatic hernia, *SD* Standard deviation. All scores are expressed as mean (SD)

^a^All 7 patients with CDH and neurologic issues had severe cognitive impairment; 1 patient also had cerebral palsy

^*^The given health-related quality of life score was compared across groups using analysis of variance (ANOVA)

**Table 4:** Child-Reported Quality of Life Scores of Patients With CDH Stratified by Characteristics at Birth, CDH Severity, and Major Lifetime Comorbid Conditions

Characteristic	Patients (n)	Mean Overall Score (SD)	*P* Value*	Mean Physical Score (SD)	*P* Value*	Mean Psychosocial Score (SD)	*P* Value*
Characteristics at birth							
Sex			0.56		0.16		0.95
Male	15	76.4 (14.3)		78.8 (17.4)		75.2 (14.2)	
Female	13	73.3 (13.7)		70.0 (14.5)		75.6 (15.6)	
Probability of survival score			0.71		0.89		0.63
Low or intermediate	8	73.4 (14.7)		75.4 (14.1)		73.2 (16.8)	
High	20	75.6 (13.9)		74.4 (17.6)		76.3 (13.9)	
CDH type			0.81		0.42		0.93
Liver down	22	75.3 (13.6)		76.0 (15.8)		75.3 (14.2)	
Liver up	6	73.7 (16.2)		69.8 (19.4)		75.8 (17.3)	
BPD			0.56		0.76		0.47
Yes	9	72.7 (12.0)		73.3 (15.6)		72.4 (11.9)	
No	19	76.0 (14.9)		75.3 (17.2)		76.8 (15.8)	
Initial hospitalization, days			0.48		0.47		0.40
≤ 30	15	76.3 (13.0)		73.8 (15.7)		78.1 (14.0)	
31–60	10	75.9 (15.7)		78.8 (18.3)		74.3 (15.1)	
≥ 61	3	65.6 (12.2)		65.6 (14.3)		65.6 (16.2)	
Mechanical ventilation, days			0.99		0.93		0.88
≤ 7	13	75.3 (16.1)		73.3 (15.7)		76.9 (17.8)	
8–21	10	74.3 (13.3)		75.6 (19.0)		73.7 (12.1)	
≥ 22	5	75.4 (11.2)		76.3 (16.3)		75.0 (11.7)	
Postdischarge events							
Hospitalizations, n			0.75		0.24		0.39
0	6	78.3 (8.5)		70.3 (14.4)		82.7 (9.8)	
1–2	11	75.3 (18.1)		81.3 (17.3)		72.7 (18.4)	
≥ 3	11	72.8 (12.0)		70.5 (15.8)		74.1 (11.9)	
Characteristics at testing							
Age, years			0.76		0.38		0.99
5–12	16	74.3 (13.8)		72.3 (16.6)		75.4 (14.4)	
≥ 13	12	75.9 (14.6)		77.9 (16.5)		75.4 (15.4)	
Digestive issues			0.22		0.51		0.18
Yes	17	72.3 (15.5)		73.0 (16.9)		72.4 (16.3)	
No	11	79.1 (10.2)		77.3 (16.2)		80.0 (10.4)	
Respiratory issues			0.02		0.01		0.10
Yes	16	69.9 (14.7)		68.0 (16.4)		71.4 (16.2)	
No	12	81.7 (9.1)		83.6 (11.9)		80.7 (10.3)	
Orthopedic issues			0.76		0.97		0.67
Yes	5	76.7 (10.5)		74.4 (17.9)		78.0 (8.0)	
No	23	74.6 (14.7)		74.7 (16.6)		74.8 (15.7)	
Neurologic issues			0.13		0.27		0.11
Yes	3	63.4 (15.8)		64.6 (15.7)		62.8 (20.0)	
No	25	76.4 (13.3)		75.9 (16.4)		76.9 (13.5)	

*BPD* Bronchopulmonary dysplasia, *CHD* Congenital diaphragmatic hernia, *SD* Standard deviation. All scores are expressed as mean (SD)

^a^The given health-related quality of life score was compared across groups using analysis of variance (ANOVA)

## Discussion

Many of our patients with CDH had substantial comorbid conditions (Appendix), and these comorbid conditions may have affected HRQoL. Using PedsQL, we found that the HRQoL of the patients with CDH, as measured by the child self-reports and parent-proxy reports, was considerably lower than that of the control participants. For both parent-proxy reports and child self-reports, the magnitude of QoL reduction was consistent for the overall score and all relevant subscores. The child self-reports showed significantly lower QoL than the parent-proxy reports; therefore, when interpreting QoL in CDH survivors, a proxy report should not be considered a substitute for a child’s self-report. This finding should be considered in situations when only proxy HRQoL report is available for decision-making [31]. In addition, discordance was found between patients with CDH and their parents in regard to specific characteristics associated with low HRQoL. For example, respiratory disease was the only characteristic markedly associated with low HRQoL in the child self-reports, but several characteristics—all descriptors of postnatal CDH severity—were associated with lower HRQoL when reported in the parent-proxy reports.

### Parent-proxy and child self-reports of HRQoL and characteristics associated with HRQoL

Because of the lower cognitive and language skills of young children, PedsQL includes parent-proxy reports to obtain information about children. The use of questionnaires with child and parent versions has raised questions about the level of agreement between children and their parents about child functioning. Some investigators reported poor parent-child agreement [32, 33], but others reported moderate to high agreement [34, 35]. One study used both child self-reports and parent-proxy reports to evaluate QoL of patients with CDH [18], but to our knowledge our study is the first to simultaneously obtain child self-reports and parent-proxy reports by using a validated instrument and to directly compare these sources. In our study, parent-reported HRQoL was slightly but consistently higher than child-reported HRQoL. When patient and disease characteristics were tested for potential associations with HRQoL, our findings were inconsistent between parent and child assessments. The lack of agreement among individual variables associated with child- and parent-reported HRQoL scores may be due to complex, interrelated, overlapping disease characteristics; thus, identifying a single characteristic that is consistently associated with low HRQoL is difficult. Patients with CDH may have complex comorbid conditions that contribute to low HRQoL scores in diverse ways when reported by a child versus a parent. In our study, the characteristics that are descriptors of the severity of the postnatal course were not associated with reduced HRQoL scores in the child self-reports. Similarly, Koivusalo et al. [17] observed lower than expected HRQoL in 25% of patients with CDH, but no correlation between CDH severity and HRQoL scores was reported. A single variable—chronic respiratory problems, which are often linked with multiple hospitalizations—was associated with low child self-reported HRQoL in our study. In contrast, longer initial hospitalization, postnatal BPD, severe cognitive impairment, and chronic orthopedic problems were all associated with lower HRQoL when reported by parents but not children.

Discordant perceptions of HRQoL between child self-reports and parent-proxy reports have been reported [36, 37]. In many instances, when the parent and child disagree, the evidence does not clearly show that parents overestimated or underestimated HRQoL. Glaser et al. [35] suggested that parents can appropriately report information about their child’s HRQoL, but others indicated that parents tend to rate their child’s HRQoL lower than their child [32, 33]. Davis et al. [31] suggested that “discordance in HRQoL scores between parents and children may be due to different reasoning and response styles rather than item interpretation.” In a study by Saigal et al. [38], parents were asked to rate the HRQoL of their infants with extremely low birth weight, and, despite the presence of substantial disease burden, average HRQoL was high. In our study, the scores of the parent-proxy reports were consistently higher than the scores of the child self-reports. Investigations of adult populations have shown that patients are more reliable and consistent at scoring their own QoL than their physicians [39, 40]; therefore, patient reports should be the criterion standard when this information is used for interventions and treatments. Another study assessed QoL in children with chronic pain and suggested that, although merit should be given to parent-proxy reports, the child’s own perspective should be directly solicited whenever possible [2].

### Association between age of patients with CDH and HRQoL scores

Because numerous comorbid conditions associated with CDH in early childhood resolve with age and chronic health conditions may affect a person’s subjective perception of HRQoL, self-reported HRQoL scores may be affected by the child’s age [19, 41]. Poley et al. [19] conducted a cross-sectional study and observed poor HRQoL in young patients with CDH but normal HRQoL in children older than 16 years. Poley et al. concluded that, despite considerable early morbidity, the ultimate prognosis of CDH as measured with HRQoL is favorable. In contrast, using PedsQL questionnaires, Sheikh et al. [41] reported good HRQoL scores in patients with CDH, even in younger patients (mean age, 5.5 years), regardless of CDH severity at birth. This discordance between studies may be due to the associations between CDH and various comorbid conditions (eg, cardiac, pulmonary), which have variable degrees of severity and potential to improve over time. In addition, given the cross-sectional nature of these studies, the association between QoL and age is difficult to interpret, because at the time they were surveyed for the present study, older patients had received treatments when contemporary therapeutic modalities were unavailable, resulting in survival of healthier children; therefore, these older CHD participants may have less-severe residual disease. For all of these reasons, comparison of outcomes in cross-sectional studies is associated with complexities related to unaccounted confounders.

In our study, HRQoL of the patients with CDH differed with the age of the child. Five- to 12-year-old children had considerably lower parent-reported HRQoL. A plausible, albeit speculative, explanation for this finding is that these children are more engaged in competitive school activities, and therefore parents may notice that their child does not perform at the same level as their peers. Whether this rationale would also apply to child self-reports cannot be assessed because children under the age of 5 years did not complete a questionnaire. However, it appears that a similar perception of low health status exists for 5- to 12-year-old children and their parents because both groups reported low scores (Tables 3 and 4).

### Limitations

This study is limited by its retrospective design. Evaluation of a wide range of ages with a cross-sectional study precludes the ability to precisely assess whether HRQoL changes with time. Given the rarity of CDH, the statistical power to assess differences across age groups is limited by the small number of patients in some age groups. In addition, a parent’s assessment of their child’s HRQoL may be influenced by unaccounted elements (eg, life experience, expectations for their child, physical and psychological health, socioeconomic status, sex, race and ethnicity, family relationships, and parental stress related to their child’s disease) [1, 2]. Furthermore, adaptation to disease may affect a child’s perception of HRQoL, but we cannot account for this possibility. In order to ensure that our control group was not skewed by individuals with chronic comorbidities, when selecting control participants, we excluded participants with chronic medical illness. This approach may have overestimated the HRQoL of our control participants compared with the general population.

## Conclusions

Across a wide range of ages, patients with CDH had lower self-reported HRQoL scores in the physical and psychosocial domains compared with similarly aged, healthy control participants. However, no patient characteristics, comorbid conditions, or symptoms were consistently associated with worse HRQoL in the child or parent assessments. Although we were unable to determine why children reported lower QoL scores, we believe that clinical interventions based on QoL information should primarily consider the child’s report, and that the child’s own perspective of well-being should be a criterion standard for implementation of corrective measures. Proxy responses for evaluation of QoL in CDH survivors should be used only when patient responses are not available.

## Appendix

**Table 5:** Chronic Health-Related Conditions in Patients with CDH

Patient	QoL Score		Age, Sex	Hospital Readmissions^a^ (N)	Major Health Issues or Conditions^b, c^
	Child Report	Parent Report			
1	NA	90.3	6 mo, M	1	RRI
2	NA	78.6	7 mo, M	1	DOR, DOE, RRI
3	NA	91.4	10 mo, M	0	Surgery (clubfoot), RRI
4	NA	95.0	3 mo, M	0	None
5	NA	80.6	20 mo, M	1	Oral aversion, malnourished, DOE, RRI, inguinal hernia repair
6	NA	86.0	22 mo, M	2	RRI, DOE
7	NA	82.2	23 mo, M	6	RRI, atopic dermatitis, inguinal hernia repair, orchidopexy
8	NA	66.3	16 mo, F	0	Multicystic kidney, IQ < 70
9	NA	82.1	4 y, F	7	Galactosemia, cataract surgery, RRI, IQ < 70
10	NA	95.2	3 y, M	1	Surgery (SBO, CDH reherniation)
11	NA	94	3 y, M	0	None
12	NA	100	4 y, M	5	DOE, DOR, nocturnal dyspnea, RRI
13	NA	96.4	4 y, F	0	RRI
14	NA	95.2	4 y, M	0	RRI
15	NA	89.3	4 y, M	2	RRI
16	NA	39.1	5 y, M	4	IQ < 70, hyperactivity disorder, DOE, RRI, NPF-up
17	63.0	89.1	5 y, F	0	RRI
18	73.9	84.9	5 y, F	1	RRI
19	81.8	85.9	5 y, F	0	RRI, DOE
20	78.3	82.6	5 y, F	0	None
21	45.7	83.7	5 y, M	2	RRI, asthma, IQ < 70
22	NA	35.8	7 y, M	4	IQ < 70, cerebral palsy, DOE, RRI, NPF-up, difficulty walking, surgeries (CDH reherniation, SBO)
23	76.1	53.3	7 y, F	15	Severe pectus excavatum, RRI, DOE, DOR, oral aversion, GERD, IQ < 70, NPF-up, pain ambulating, surgery (SBO)
24	62.0	72.8	8 y, F	4	DOE, RRI, hydronephrosis, surgeries (CDH reherniation, neck fistula, preauricular tag)
25	85.9	84.8	8 y, M	6	DOE, RRI, surgery (equinovarus, orchidopexy)
26	94.6	81.5	9 y, M	13	NPF-up, oral aversion, malnourished, GERD, DOE, RRI, surgeries (adenoidectomy, abdominal patch removal)
27	81.5	82.6	10 y, F	1	Surgery (SBO)
28	52.2	54.8	11 y, F	4	NPF-up, seizures, GERD, DOE, hearing and visual impairment, surgery (SBO)
29	75.0	89.1	11 y, M	3	Oral aversion, pain ambulating, selective mutism, NPF-up
30	66.3	67.3	12 y, M	1	Adenoidectomy
31	89.1	86.9	12 y, F	0	Congenital blindness, attending special school, NPF-up
32	71.7	80.0	12 y, M	5	DOE, RRI, surgeries (inguinal hernia repair, orchidopexy)
33	91.3	88.0	13 y, M	2	RRI, orchidopexy
34	68.5	92.4	13 y, M	5	NPF-up, IQ < 70, learning difficulties, vomiting, RRI, surgeries (SBO, closure ductus Botalli)
35	95.7	98.9	13 y, M	2	Surgeries (orchidopexy, hydrocele repair)
36	79.4	58.69	14 y, F	1	Brace therapy (scoliosis), pain ambulating
37	79.4	58.7	14 y, M	0	Primary night enuresis, NPF-up
38	83.7	100	14 y, M	3	Atopic asthma
39	45.7	78.3	15 y, F	2	DOE, adenoidectomy
40	64.1	70.7	15 y, M	1	GERD, chest pain
41	67.4	73.9	15 y, M	3	CVI due to AV malformation, residual hemiparesis, loss of visual field, NPF-up
42	64.1	77.2	17 y, M	4	Bronchiectasis, GERD, DOE, RRI
43	91.3	92.4	18 y, F	1	Pain ambulating
44	93.5	88.0	20 y, M	2	None
45	78.3	90.2	20 y, F	0	Hashimoto thyroiditis, DOE

*AV* Arteriovenous, *CDH* Congenital diaphragmatic hernia, *CVI* Cerebrovascular insult, *DOE* Dyspnea on exertion, *DOR* Dyspnea on rest, *F* Female, *GERD* Gastroesophageal reflux disease, *IQ* Intelligence quotient, *M* Male, *N* Number, *NA* Not applicable, *NPF-up* Neurologic and/or psychiatric follow-up, *QoL* Quality of life, *RRI* Recurrent respiratory infection, *SBO* Small-bowel obstruction

^a^Readmissions account for all hospitalizations after discharge from initial treatment; ^b^ Information was obtained from medical records and by interview; ^c^ Includes some major morbidities or surgical procedures during initial hospitalization for treatment of CDH and all comorbidities after initial discharge

## Abbreviations

BPD: Bronchopulmonary dysplasia
CDH: Congenital diaphragmatic hernia
HRQoL: Health-related quality of life
PedsQL: Pediatric Quality of Life Inventory
QoL: Quality of life
RRI: Recurrent respiratory infection

## References

[1] Grimaldi Capitello T, Fiorilli C, Placidi S, Vallone R, Drago F, Gentile S (2016). What factors influence parents’ perception of the quality of life of children and adolescents with neurocardiogenic syncope?. Health Qual Life Outcomes.

[2] Vetter TR, Bridgewater CL, McGwin G (2012). An observational study of patient versus parental perceptions of health-related quality of life in children and adolescents with a chronic pain condition: who should the clinician believe?. Health Qual Life Outcomes.

[3] Hyland ME (2003). A brief guide to the selection of quality of life instrument. Health Qual Life Outcomes.

[4] Gale CR, O'Callaghan FJ, Bredow M, Martyn CN (2006). The influence of head growth in fetal life, infancy, and childhood on intelligence at the ages of 4 and 8 years. Pediatrics.

[5] Su CT, Wang JD, Lin CY (2013). Child-rated versus parent-rated quality of life of community-based obese children across gender and grade. Health Qual Life Outcomes.

[6] Bojanic K, Pritisanac E, Luetic T, Vukovic J, Sprung J, Weingarten TN (2015). Survival of outborns with congenital diaphragmatic hernia: the role of protective ventilation, early presentation and transport distance: a retrospective cohort study. BMC Pediatr.

[7] Bojanic K, Pritisanac E, Luetic T, Vukovic J, Sprung J, Weingarten TN (2015). Malformations associated with congenital diaphragmatic hernia: impact on survival. J Pediatr Surg.

[8] Bojanic K, Grizelj R, Dilber D, Saric D, Vukovic J, Pianosi PT (2016). Cardiopulmonary exercise performance is reduced in congenital diaphragmatic hernia survivors. Pediatr Pulmonol.

[9] Bojanic K, Grubic M, Bogdanic A, Vukovic J, Weingarten TN, Huebner AR (2016). Neurocognitive outcomes in congenital diaphragmatic hernia survivors: a cross-sectional prospective study. J Pediatr Surg.

[10] Bouman NH, Koot HM, Tibboel D, Hazebroek FW (2000). Children with congenital diaphragmatic hernia are at risk for lower levels of cognitive functioning and increased emotional and behavioral problems. Eur J Pediatr Surg.

[11] Brownlee EM, Howatson AG, Davis CF, Sabharwal AJ (2009). The hidden mortality of congenital diaphragmatic hernia: a 20-year review. J Pediatr Surg.

[12] Nobuhara KK, Lund DP, Mitchell J, Kharasch V, Wilson JM (1996). Long-term outlook for survivors of congenital diaphragmatic hernia. Clin Perinatol.

[13] Peetsold MG, Heij HA, Kneepkens CM, Nagelkerke AF, Huisman J, Gemke RJ (2009). The long-term follow-up of patients with a congenital diaphragmatic hernia: a broad spectrum of morbidity. Pediatr Surg Int.

[14] Peetsold MG, Huisman J, Hofman VE, Heij HA, Raat H, Gemke RJ (2009). Psychological outcome and quality of life in children born with congenital diaphragmatic hernia. Arch Dis Child.

[15] Peetsold MG, Vonk-Noordegraaf A, Heij HH, Gemke RJ (2007). Pulmonary function and exercise testing in adult survivors of congenital diaphragmatic hernia. Pediatr Pulmonol.

[16] Chen C, Jeruss S, Chapman JS, Terrin N, Tighiouart H, Glassman E (2007). Long-term functional impact of congenital diaphragmatic hernia repair on children. J Pediatr Surg.

[17] Koivusalo A, Pakarinen M, Vanamo K, Lindahl H, Rintala RJ (2005). Health-related quality of life in adults after repair of congenital diaphragmatic defects--a questionnaire study. J Pediatr Surg.

[18] Michel F, Baumstarck K, Gosselin A, Le Coz P, Merrot T, Hassid S (2013). Health-related quality of life and its determinants in children with a congenital diaphragmatic hernia. Orphanet J Rare Dis.

[19] Poley MJ, Stolk EA, Tibboel D, Molenaar JC, Busschbach JJ (2004). Short term and long term health related quality of life after congenital anorectal malformations and congenital diaphragmatic hernia. Arch Dis Child.

[20] Boloker J, Bateman DA, Wung JT, Stolar CJ (2002). Congenital diaphragmatic hernia in 120 infants treated consecutively with permissive hypercapnea/spontaneous respiration/elective repair. J Pediatr Surg.

[21] Downard CD, Jaksic T, Garza JJ, Dzakovic A, Nemes L, Jennings RW (2003). Analysis of an improved survival rate for congenital diaphragmatic hernia. J Pediatr Surg.

[22] Frenckner B, Ehren H, Granholm T, Linden V, Palmer K (1997). Improved results in patients who have congenital diaphragmatic hernia using preoperative stabilization, extracorporeal membrane oxygenation, and delayed surgery. J Pediatr Surg.

[23] Grizelj R, Bojanic K, Pritisanac E, Luetic T, Vukovic J, Weingarten TN (2016). Survival prediction of high-risk outborn neonates with congenital diaphragmatic hernia from capillary blood gases. BMC Pediatr.

[24] Grizelj R, Bojanic K, Vukovic J, Novak M, Rodin U, Coric T (2016). Epidemiology and outcomes of congenital diaphragmatic hernia in Croatia: a population-based study. Paediatr Perinat Epidemiol.

[25] Varni JW. PedsQL [http://www.pedsql.org]. Accessed 11 Sept 2017.

[26] Hill CD, Edwards MC, Thissen D, Langer MM, Wirth RJ, Burwinkle TM (2007). Practical issues in the application of item response theory: a demonstration using items from the pediatric quality of life inventory (PedsQL) 4.0 generic core scales. Med Care.

[27] Varni JW, Limbers CA, Burwinkle TM (2007). Impaired health-related quality of life in children and adolescents with chronic conditions: a comparative analysis of 10 disease clusters and 33 disease categories/severities utilizing the PedsQL 4.0 generic Core scales. Health Qual Life Outcomes.

[28] Jobe AH, Bancalari E (2001). Bronchopulmonary dysplasia. Am J Respir Crit Care Med.

[29] Congenital Diaphragmatic Hernia Study Group (2001). Estimating disease severity of congenital diaphragmatic hernia in the first 5 minutes of life. J Pediatr Surg.

[30] Schaad UB (2010). OM-85 BV, an immunostimulant in pediatric recurrent respiratory tract infections: a systematic review. World J Pediatr.

[31] Davis E, Nicolas C, Waters E, Cook K, Gibbs L, Gosch A (2007). Parent-proxy and child self-reported health-related quality of life: using qualitative methods to explain the discordance. Qual Life Res.

[32] Ennett ST, DeVellis BM, Earp JA, Kredich D, Warren RW, Wilhelm CL (1991). Disease experience and psychosocial adjustment in children with juvenile rheumatoid arthritis: children's versus mothers’ reports. J Pediatr Psychol.

[33] Theunissen NC, Vogels TG, Koopman HM, Verrips GH, Zwinderman KA, Verloove-Vanhorick SP (1998). The proxy problem: child report versus parent report in health-related quality of life research. Qual Life Res.

[34] Varni JW, Limbers C, Burwinkle TM (2007). Literature review: health-related quality of life measurement in pediatric oncology: hearing the voices of the children. J Pediatr Psychol.

[35] Glaser AW, Davies K, Walker D, Brazier D (1997). Influence of proxy respondents and mode of administration on health status assessment following central nervous system tumours in childhood. Qual Life Res.

[36] Lim Y, Velozo C, Bendixen RM (2014). The level of agreement between child self-reports and parent proxy-reports of health-related quality of life in boys with Duchenne muscular dystrophy. Qual Life Res.

[37] Upton P, Lawford J, Eiser C (2008). Parent-child agreement across child health-related quality of life instruments: a review of the literature. Qual Life Res.

[38] Saigal S, Rosenbaum PL, Feeny D, Burrows E, Furlong W, Stoskopf BL (2000). Parental perspectives of the health status and health-related quality of life of teen-aged children who were extremely low birth weight and term controls. Pediatrics.

[39] Schnadig ID, Fromme EK, Loprinzi CL, Sloan JA, Mori M, Li H (2008). Patient-physician disagreement regarding performance status is associated with worse survivorship in patients with advanced cancer. Cancer.

[40] Slevin ML, Plant H, Lynch D, Drinkwater J, Gregory WM (1988). Who should measure quality of life, the doctor or the patient?. Br J Cancer.

[41] Sheikh F, Akinkuotu A, Clark SJ, Zamora IJ, Cass DL, Olutoye O (2016). Assessment of quality of life outcomes using the pediatric quality of life inventory survey in prenatally diagnosed congenital diaphragmatic hernia patients. J Pediatr Surg.

